# Role of MicroRNA in Governing Synaptic Plasticity

**DOI:** 10.1155/2016/4959523

**Published:** 2016-03-13

**Authors:** Yuqin Ye, Hongyu Xu, Xinhong Su, Xiaosheng He

**Affiliations:** ^1^Department of Neurosurgery, Xijing Hospital, Fourth Military Medical University, Xi'an 710032, China; ^2^Department of Neurosurgery, Second Affiliated Hospital of Hunan Normal University (PLA 163 Hospital), Changsha 410000, China

## Abstract

Although synaptic plasticity in neural circuits is orchestrated by an ocean of genes, molecules, and proteins, the underlying mechanisms remain poorly understood. Recently, it is well acknowledged that miRNA exerts widespread regulation over the translation and degradation of target gene in nervous system. Increasing evidence suggests that quite a few specific miRNAs play important roles in various respects of synaptic plasticity including synaptogenesis, synaptic morphology alteration, and synaptic function modification. More importantly, the miRNA-mediated regulation of synaptic plasticity is not only responsible for synapse development and function but also involved in the pathophysiology of plasticity-related diseases. A review is made here on the function of miRNAs in governing synaptic plasticity, emphasizing the emerging regulatory role of individual miRNAs in synaptic morphological and functional plasticity, as well as their implications in neurological disorders. Understanding of the way in which miRNAs contribute to synaptic plasticity provides rational clues in establishing the novel therapeutic strategy for plasticity-related diseases.

## 1. Introduction

Synaptic plasticity, as a specific form of neural plasticity, not only plays an important role in maintaining neural physiological function but also contributes to many nervous system diseases including neurotrauma, neurodegenerative disease, and mental disorder [1, 2]. Actually, synapse structural plasticity and functional plasticity are collectively referred to as the term of synaptic plasticity. The structural plasticity covers the changes of synaptic morphology and number in adapting to activity-induced neural network variation, including dendritic spine modification, axonal sprouting, and new synaptic formation [3]. The functional plasticity encompasses the alterations of synaptic transmission and efficacy in response to neural activity, such as long-term potentiation (LTP), long-term depression (LTD), and homeostatic plasticity [3, 4]. In recent studies, it has been shown that numerous molecules and genes are involved in the complex regulatory process of synaptic plasticity, through which the function of nervous system is coordinated and maintained.

MicroRNA (miRNA), an extensive class of evolutionarily conserved noncoding RNA, contains approximate 22 nucleotides and is involved in post-transcriptional modulation of gene expression. They function collectively to direct mRNA translational inhibition and degradation and to coordinate many physiological and pathophysiological signaling pathways [5]. More than half of those detectable miRNAs are abundant in mammalian nervous system, and accumulating evidence points to a widespread involvement of miRNA in neural development and function [6–8]. Notably, individual miRNAs are particularly enriched in presynaptic and postsynaptic compartments, where they serve to regulate synaptic plasticity and activity by coordinating the intricate genetic circuitries [9, 10]. In this review, we aim to analyze the current data regarding the function of individual miRNAs in synaptic plasticity and, in particular, to understand their regulatory role in synapse morphological and functional plasticity, as well as their implications in plasticity-related neurological diseases.

## 2. Overview of miRNA Biogenesis and Action

**Figure 1:**
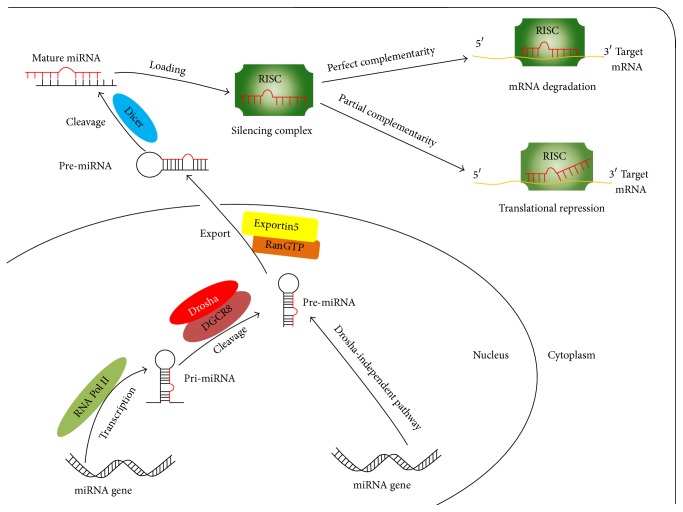
Biogenesis and action of miRNA.

The general biogenesis and diverse functions of miRNA are increasingly becoming clear (Figure 1). There is a series of precise and consecutive processing steps in* Drosha*-dependent pathways of miRNA biogenesis. Initially, the transcription of primary miRNA (pri-miRNA) is mediated by RNA polymerase II in nucleus. Secondly, in the RNase III-family endonuclease* Drosha*/*DiGeorge* syndrome critical region 8 (DGCR8) dependent pathway, the pri-miRNA is cleaved into characteristic precursor miRNA (pre-miRNA), which has a hairpin-like long stem-loop structure containing about 70 nucleotides [11]. Next, pre-miRNA is exported from nucleus into cytoplasm via Exportin5/RanGTP machinery. Then, a second round of processing is mediated by the enzyme Dicer, which cleaves the pre-miRNA into a miRNA duplex of 22 nucleotides in cytoplasm; after that, the duplex is unwound by a helicase. One strand of the duplex interacts with its partner proteins such as argonaute family proteins (AGO2 in* Drosophila* and eIF2C in human) before they are processed into the RNA-induced silencing complex (RISC) [12]. Afterwards, the RISC complex incorporates with mature miRNA to form dissymmetric ribonucleoproteins complex, which will bind to target mRNA in 3′ untranslated region (UTR) through the complementary sequence including 6 to 8 nucleotides and thereby act on mRNA degradation or translational repression [7, 13]. In addition, miRNA is able to prevent circularization and inhibit translational initialing of m7G-capped mRNAs by targeting its 5′ cap region [14, 15].

However, besides the canonical mechanism for biogenesis of most miRNAs, alternative* Drosha*-independent pathways are involved in the synthesis and maturation of some specific miRNA populations [16–18]. One of the widely studied pathways is splicing-mediated pathway in the biogenesis of introns-derived miRNA (known as “miRtron”), which is originally identified in* Drosophila *and* Caenorhabditis elegans* [17, 18]. Following encoding in the introns of protein coding genes, miRtron is spliced and debranched, respectively, by spliceosome and lariat debranching enzyme, after which it is endowed with the features resembling those of pre-miRNA and turns into the main stream miRNA biogenesis pathway to produce functional miRNA* in vivo* [18, 19]. miRtrons are not only identified in invertebrate but also recognized recently in mammal such as mouse and human [17, 20, 21]. Interestingly, Havens et al. reported that two presumable human miRtrons did not rely on the splicing-mediated pathway and thereby termed them as splicing-independent miRtrons (simtrons), but the maturation of the two simtrons was inhibited by dominant negative form of* Drosha* in canonical pathway* in vitro* [17]. Hence, it can be inferred that the underlying cross talk among different pathways may exist and collectively act on the biogenesis of these distinct miRNAs* in vivo*; further exploration is required to elucidate it.

Both canonical and noncanonical pathways of miRNA biogenesis lay foundation for uncovering the interactions between miRNAs and synaptic plasticity. Although the notion that involvement of miRNAs in synaptic plasticity is quite recently coming up, the multiple role and mechanism have been investigated by extensive studies. Most recently, growing investigations have shed light on the sophisticated roles of specific miRNAs in modification of plasticity-related proteins within postsynaptic density, which benefits us to understand the influencing factors and underlying mechanism of synaptic plasticity [9, 22]. Based on the previous studies, it is possible that individual miRNAs are versatile processors in governing synapse morphological and functional plasticity, as stated below.

## 3. Regulation of miRNA-132 in Synaptic Plasticity

### 3.1. miRNA-132 and cAMP-Response Element Binding Protein (CREB)

miRNA-132 is the most studied plasticity-related miRNA with diverse functions in the processing of synapse plasticity. The CREB induced miRNA-132 is capable of regulating neuronal morphogenesis and synaptic protein synthesis in response to neurotrophin* in vitro* [23, 24]. The fact that activation of neural networks in olfactory bulb, hippocampus, and striatum induced a rapid and significant rise of CREB-regulated miRNA-132* in vivo *was reported for the first time by Nudelman et al. in 2010 [25]. Accordingly, both* in vitro *and* in vivo *determinations implicate that miRNA-132 is an activity-dependent miRNA and thereby contributes to experience-induced synapse proteomic expression that is regarded as necessary for synaptic plasticity [23–25]. Similarly, the findings are further confirmed and extended by detection of miRNA-132/phosphorylated-CREB (p-CREB) signaling chain in animals and patients with temporal lobe epilepsy (TLE). Compared with sham, the levels of p-CREB and miRNA-132 in epilepsy were significantly upregulated at 24 h after seizure that was characterized with excitatory synaptic activity, new synaptic connections, and LTP in hippocampus [26]. Correspondingly, microRNA-132 silencing inhibits the mossy fiber sprouting (MFS) and dendritic morphology in hippocampal CA3 region, which results in suppression of spontaneous recurrent seizures [27]. Therefore, it is possible that miRNA-132 plays a pathogenic role in epilepsy by processing the synapse activity-dependent plasticity, and intervention of the plasticity will be a novel therapeutic target for epilepsy. In addition, the CREB-regulated miRNA-132 also serves as a dynamic activity-dependent processor of cognition capacity; the level of miRNA-132* in vivo* is fine-tuned within a suitable range to improve synaptic plasticity for learning and memory [28, 29].

### 3.2. miRNA-132 and Brain-Derived Neurotrophic Factor (BDNF)

It is extensively characterized that BDNF, a critical member of the neurotrophin family, exerts multiple effects on synapse via activation of various intracellular signaling pathways such as phosphoinositide-3-kinase (PI3K), phospholipase C-*γ* (PLC-*γ*), mitogen-activated protein kinase/extracellular signal regulated protein kinase (MAPK/ERK), and CREB [24, 30–33]. However, the presumable involvement of miRNAs in BDNF-regulated synaptic function is less well revealed. The interplay between miRNA-132 and BDNF is a focus of recent interests. miRNA-132 is upregulated dose-dependently by BDNF to promote synaptic formation and plasticity [24, 30]. Kawashima and associates demonstrate that BDNF affects miRNA-132 level through MAPK/ERK1/2 pathway to influence synthesis of postsynaptic protein. Glucocorticoid exposure also results in a decrease in BDNF-induced synaptic function via inhibiting expression of miRNA-132 [30]. Furthermore, CREB is available for positive modulation of memory performance and consolidation by regulating the expression of BDNF [23]. In addition, new insight into miRNA-132 action has been verified in the development of retinal ganglion cell (RGC) axon. In this study, BDNF treatment gives rise to significant upregulation of miRNA-132 in RGC, which results in promotion of axon sprouting and growth [34].

### 3.3. miRNA-132 and p250 GTPase Activating Protein (p250GAP)

As a downstream target of miRNA-132, p250GAP knockdown not only enhances hippocampal activity-dependent neuronal morphogenesis and the frequency of miniature excitatory postsynaptic currents (mEPSCs) but also promotes dendritic spine formation and the prevalence of glutamate receptor 1 (GluR1) positive spines. Yet upregulation of p250GAP by inhibiting the expression of miRNA-132 can give rise to a converse effect [35, 36]. Moreover, p250GAP is responsible for the activation of Rac1/Pak cascade, which has been identified as another downstream of miRNA-132 and specifically contributes to dendritic spine formation [36]. Shaltiel et al. provided further evidences that extended miRNA-132 to inhibit the level of hippocampal acetylcholinesterase (AChE), which played an important role in stressful experience-induced neurite extension and cognitive function [37]. In another example, cultured hippocampal HT22 cells suffering from ionizing radiation enabled a reduction of miRNA-132 and Rac1 in miRNA-132/p250GAP/Rac1/Cofilin signaling pathway, which was closely related to synaptic actin-remodeling and spine morphological alterations that enabled correct processing of learning and memory. In line with the* in vitro *result, the* in vivo *detection further confirmed the effect of miRNA-132 on irradiated mice brain [38]. Intriguingly, the powerful regulator of energy homeostasis, leptin, can act in hippocampus to promote the formation of stable dendritic spines and functional synapses by inducing CREB transcription and increasing miRNA-132-mediated suppression of p250GAP activity, which eventually improve cognitive function and attenuate depression or anxiety [39]. Additionally, in the neurotoxicity of polychlorinated biphenyls (PCB95), the sensitized ryanodine receptors (RyR) can upregulate miRNA-132 expression via CREB-dependent mechanism and subsequently inhibit the translation of p250GAP, resulting in abnormal enhancement of neuronal connectivity, aberrant synaptogenesis, incorrect synaptic network, and neuropsychological dysfunction [40]. Therefore, it can be concluded that miRNA-132/p250GAP pathway and its cross talk with other cascades play a significant role in the regulation of synapse plasticity and that inhibiting this pathway may be beneficial to plasticity-related neurological diseases.

### 3.4. miRNA-132 and Methyl CpG-Binding Protein 2 (MeCP2)

Another negative-regulated target of miRNA-132 is MeCP2, which is enriched in developing central nervous system (CNS) and highly correlated with activity-induced synaptic plasticity and axonal and dendritic development [41]. In the hippocampus of miRNA-132 overexpressed mice, significantly decreased MeCP2 level and increased dendritic spine density were observed, while the novel object recognition memory of these mice was impaired [42]. Meanwhile, the expression of MeCP2 and the functions of spatial learning and memory were also affected in miRNA-132/212 knockout mice [43]. Indeed, MeCP2 mutation restricts synaptogenesis, dendritic spine maturation, and neural circuits development, which is characterized in the pathogenesis of most Rett syndrome (RTT) cases and is closely linked to the mental abnormalities of RTT [44, 45]. Genome-wide screening showed that the expressions of miRNA-132 and BDNF were significantly decreased in a mouse model of RTT, suggesting that dysregulation of miRNA-132 was involved in the progression of RTT and boosting the level of miRNA-132 might be a valuable strategy for RTT therapy [46].

Most recently, the study by Zhang et al. stated that 2.5-fold overexpression of MeCP2 was available for attenuating both acute pain transduction and chronic pain formation in spinal cord via miR-132/CREB pathway, but excessive high level of MeCP2 would cause abnormal axonal arborization and had no analgesic role in pain transmission [47]. Therefore, this data indicates that intervention of miRNA-132/MeCP2 cascade to maintain MeCP2 within a proper and narrow range may be a promising therapeutic option for spinal cord pain. Further study revealed a modulatory feed loop for the homeostatic regulation of MeCP2 and miRNA-132* in vivo* [48]. In the loop circuit, besides the known negative regulation of MeCP2 by miRNA-132, MeCP2 also plays a feedback control role in the transcription of miRNA-132 [46, 48].

### 3.5. miRNA-132 and Fragile X Mental Retardation Protein (FMRP)

It has been showed that miRNA-132 and the proteins (e.g., Dicer and AGO1) of RISC could biochemically interact with FMRP, which was involved in regulation of synaptic plasticity by repressing translation of its mRNA ligands and synthesis of proteins at the synapse [49, 50]. Deletion of FMRP could result in pronounced overgrowth of dendritic spine and abnormal synthesis of synaptic protein at the larval neuromuscular junction (NMJ) [49]. Additionally, in the regulation of dendritic spine morphology by miRNA-132 and miRNA-125b, the downstream targets of the two miRNAs are tightly associated with FMRP to encode plasticity-related proteins involved in synaptic function [51]. Another recent study identified that FMRP along with the candidate RNA-binding protein Ataxin-2 modulated long-term plasticity via common presynaptic and postsynaptic target mRNA [52]. Since the abnormalities of synaptic plasticity resulting from FMRP dysfunction are known pathogenesis of fragile X syndrome (FXS) [53], therefore, it could be hypothesized that FXS might be, at least in part, related to the dysfunction of miRNA-132, yet the definite mechanism remains to be clarified.

### 3.6. miRNA-132 and miRNA-212

As miRNA-132 and miRNA-212 derive from the same intron of a small noncoding gene, they are capable of sharing the same targets and act in a common manner on synaptic plasticity [54]. It has been demonstrated that the expression pattern of pri-miRNA-132/212 and pre-miRNA-132/212 are affected in parallel during the process of dentate gyrus LTP [55]. Ablation of miRNA-132/212 can inhibit the neocortical *θ* burst-induced LTP and hippocampal synaptic transmission, which is possibly ascribed to the decreased postsynaptic *α*-amino-3-hydroxy-5-methyl-4-isoxazolepropionic acid receptor (AMPAR) [56]. With the analysis of bidirectional ratio metric miRNA sensors in hippocampus, Magill and colleagues also revealed that deletion of the miRNA-132/212 locus led to a dramatic decrease in dendritic length, arborization, and spine density of hippocampal newborn neurons [57]. Moreover, in the processing of hippocampal functional synapse formation, miRNA-132 is the key functional generation of the miRNA-132/212 locus to mediate the integration of newborn neurons into the dentate gyrus and contributes to the dendritic phenotype [57, 58]. In addition, the repressor element sites in promoter regions of miRNA-132 can be functionally targeted by repressor element 1 silencing transcription factor/neuron-restrictive silencing factor (REST/NRSF), and this interaction is essential for hippocampal newborn neurons activity and structural remodeling [59].

Interestingly, miRNA-132/212 also act on the synaptic plasticity of visual cortex [60–62]. It has been found that monocular deprivation would be in a position to hinder the modification of miRNA-132/212 locus and subsequently decreased the miRNA-132/212 cluster transcription in developing visual cortex [60]. Likewise, parallel findings revealed that the expression of miRNA-132 in the visual cortex was upregulated by light stimulation and delayed in response to dark-rearing. Inhibition of miRNA-132 can prevent dendritic spine maturation, ocular dominance plasticity, and formation of synaptic connectivity* in vivo* [61, 62].

## 4. Regulation of miRNA-134 in Synaptic Plasticity

### 4.1. miRNA-134 and Sirtuin1 (SIRT1)

Another brain-enriched miRNA that has been found to be involved in the regulation of synaptic plasticity is miRNA-134. In the processing of miRNA-134 transport to dendrites, Bicker et al. showed that DEAH-box helicase DHX36 was responsible for the dendritic localization of miRNA-134 and the modification of miRNA-134 in dendritic spine size [63]. Furthermore, Gao and associates found that deacetylase SIRT1 was able to suppress the expression of miRNA-134 via cooperating with the transcription factor Yin Yang 1 (YY1) and additional proteins [64]. In their study, overexpression of miRNA-134 in hippocampal CA1 region following SIRT1 deficiency led to a decreased level of CREB and BDNF and thereby impairing LTP and long-term memory formation in contextual fear conditioning [64]. Conversely, activation of SIRT1 could rescue the damaged synaptic plasticity by inhibiting the expression and posttranscriptional regulation of miRNA-134 [64]. In addition, miRNA-134 has also been implicated in the regulation of activity-induced dendritic excitability and outgrowth, as well as the number of excitatory synapses in hippocampus [65].

### 4.2. miRNA-134 and Lim Kinase 1 (Limk1)

Overexpression of miRNA-134 causes a marked reduction in the dendritic spine volume and synaptic strength of hippocampal neurons* in vitro*, whereas silencing of endogenous miRNA-134 results in an increased spine width and enhanced synaptic transmission [66]. Another cortical morphometric observation revealed that overexpression of miRNA-134 in brain could reduce the dendritic arborization of cortical layer V pyramidal neurons* in vivo *[67]. Further mechanic analysis identified that these effects were mediated by the repression of miRNA-134 on Limk1 translation, which was critical to the regulation of Cofilin phosphorylation and actin dynamics for synaptic function and plasticity [66, 68, 69]. Accordingly, it might raise the possibility that miRNA-134 worked as a negative modulator of dendritic spine development, synaptic formation, and plasticity. Moreover, it has been demonstrated that BDNF has the potential to relieve miRNA-134 inhibition of Limk1 through activation of the TrkB/mTOR pathway and thereby results in the reversible effect [66, 70, 71]. However, some studies indicated that miRNA-134 activity was restricted to specific neurons such as inhibitory GABAergic interneurons rather than other types of cortical neurons including pyramidal neurons. In these interneurons, the palmitoylation enzyme DHHC9 serves as a putative target of miRNA-134 and is critical for the modification of H-Ras, which displays a pivotal role in neuronal membrane trafficking and LTP modification [72, 73].

Studies focusing on the association between miRNA and epilepsy revealed that hippocampal miRNA-134 level was significantly upregulated in TLE [74, 75]. Silencing of miRNA-134 is able to decrease the expression of Limk1 and leads to unexpectedly reduced pyramidal neuron spine density and improved seizures, but no alteration in dendritic spine size [74]. However, a subsequent study identified that miRNA-134 remained at a low level while the expressions of CREB and p-CREB were significantly increased in hippocampus of epileptic rats [76]. Taken together, these data suggest that miRNA-134 is implicated in regulation of synaptic plasticity in epilepsy through diverse downstream targets such as Limk1 and CREB. However, further investigations are expected to clarify the possible cross talk and collective effect of different signaling cascades.

## 5. Regulation of miRNA-138 in Synaptic Plasticity

### 5.1. miRNA-138 and Acyl Protein Thioesterase1 (APT1)

miRNA-138 is ubiquitous with high expression in the dendrites of hippocampal neurons and contributes to regulation of dendritic spine size and structure [77, 78]. Functional screening demonstrates that APT1-induced palmitoylation of G protein *α*13 (G*α*13) is important for the regulatory function of miRNA-138 during dendritic spine development [77]. High level of miRNA-138 leads to a significant decrease in APT1 level and subsequently increases G*α*13 palmitoylation and membrane localization, which can activate the Rho-dependent signaling cascade and thereby induce dendritic spine shrinkage with a concomitant reduction in synaptic transmission [77].

Recently, a panel of studies focusing on miRNA-138 function during the process of learning and memory showed a close association with local plasticity-related protein synthesis [79, 80]. According to the detection of human postmortem brain tissue, miRNA-138 and decapping mRNA 1B (DCP1B) were identified to express in hippocampus and frontal cortex. Furthermore, Schröder et al. found that a human memory-associated single nucleotide polymorphism could interfere with miRNA-138 binding to the transcripts of DCP1B, implying that miRNA-138 might be a modulator of human memory performance [79]. In another determination, high level of miRNA-138 and its suppression on downstream target APT1 mRNA translation in the CA1 and DG give rise to better performance on novel object recognition task that reflects the short-term recognition memory [80]. Intriguingly, the hippocampal APT1 mRNA is dramatically increased in aged mice, but no alteration occurs in the status of impaired memory. And, in particular, the expression of APT1 protein presents location-specific pattern; its level in neuropil is evidently higher than that in cell body of neurons [80].

### 5.2. miRNA-138 and Sirtuin1 (SIRT1)

miRNA-138 binding directly to the 3′ UTR of SIRT1 mRNA results in a marked decrease in the expression of SIRT1 and a significant inhibition of axon extension in DRG neurons, whereas overexpression of SIRT1 can dramatically promote axon growth [81]. This result indicates that SIRT1 serves as a downstream functional target of miRNA-138 in the modification of intrinsic axon growth capacity. In addition, SIRT1 is capable of acting as a feedback inhibitory factor to repress the level of miRNA-138* in vivo*, suggesting that the interplay of miRNA-138 and SIRT1 is likely to be an effective intervening target to enhance axon regeneration [81].

## 6. Regulation of miRNA-9 in Synaptic Plasticity

miRNA-9 is one of the synapse-enriched miRNAs regulating gene expression. Using the miRNA sponge technique to silence the miRNA-9 conditionally in mice, Giusti et al. discovered that hippocampal dendrites growth and synaptic transmission were disturbed and concomitant with strong upregulation of transcriptional repressor REST. Further determination revealed that shRNAs against REST could rescue the dendritic growth deficit and impair synaptic transmission caused by miRNA-9 depletion [82]. Hence, it is possible that miRNA-9 is essential for synaptic formation and plasticity by targeting REST* in vivo*. Moreover, another study defined a novel role of miRNA-9 and its predictable target forkhead box G 1 (FOXG1) in olfactory receptor neurons differentiation, axon elongation, and synaptic connectivity [83]. Meanwhile, the expression of miRNA-9 and miRNA-200 was regulated by transcriptional factor distal-less homeobox5 (DLX5) [83]. Additionally, two FMRP homologous fragile X related (FXR) proteins, FXR1P and FXR2P, are believed to participate in RNA metabolism [84]. Indeed, the level of miRNA-9 and miRNA-124 decreased in the brain of FXR1P knockout mouse, and overexpression of FXR1P led to significantly increased expression of miRNA-9 and miRNA-124. However, absence of either FXR2P or FMRP does not affect level of the two brain-specific miRNAs [85]. Accordingly, the data indicated that both miRNA-9 and miRNA-124 might not be responsible for FXS that mainly resulted from the loss of FMRP.

Amyloid *β*42- (A*β*42-) dependent downregulation of miRNA-9 provides novel insight into amyloidogenesis in the pathological process of Alzheimer's disease (AD) [86]. It has been documented that A*β*42 oligomers induce dendritic spine loss and microtubule-associated protein tau hyperphosphorylation by the calcium/calmodulin-dependent protein kinase kinase 2, adenosine monophosphate-activated protein kinase (CAMKK2-AMPK) signaling cascade, which is tightly associated with synaptic plasticity alteration in AD progression [87, 88]. Overexpression of miRNA-9 could attenuate the A*β*-induced synaptotoxic effect via inhibition of CAMKK2 level, implying that CAMKK2 emerged as an additional target of miRNA-9 in the abnormal synaptic plasticity of AD [89]. In addition, tau hyperphosphorylation in the progression of AD is also promoted by upregulated miRNA-138 via retinoic acid receptor alpha/glycogen synthase kinase-3*β* (RARA/GSK-3*β*) pathway [90]. However, whether or not and how synaptic plasticity responds to the increased tau phosphorylation in AD have not been identified, and future investigation is required to elucidate it.

## 7. Regulation of miRNA-124 in Synaptic Plasticity

Another highly and specifically expressed miRNA in nervous system is miRNA-124, which contributes to a multitude of biological processes such as neurogenesis, synapse morphology, and synaptosome transmission [91, 92]. Recently, miRNA-124 is reported to play an extending role in inhibiting the expression of RhoG [93]. This interaction is further involved in regulating the actin cytoskeleton in plasticity of synapse connections, which refers to dual modes: on one hand, RhoG is available to inhibit dendritic tree complexity via the small GTPase Cdc42 in hippocampal neuron; on the other hand, RhoG is pointed out to repress axonal branching and targeting dependent on the ELMO/Dock180/Rac1 signaling pathway [93–95]. Several groups have identified that the intelligence quotient motif containing GTPase activating protein 1 (IQGAP1), a broadly expressed scaffold protein in brain, acted as a suppressed target of miRNA-124 to regulate hippocampal LTP, long-term memory formation, and cognitive performance [96, 97]. Moreover, in cultured neurons of* Aplysia *sensorimotor synapses, miRNA-124 is localized exclusively to the presynaptic sensory neuron but found deficiently in motor neuron. Overexpression of miRNA-124 inhibits the serotonin-triggered synaptic long-term facilitation (LTF) by targeting CREB, and downregulation of miRNA-124 reversely gives rise to significant enhancement of LTF in learning-related synaptic plasticity [92]. In addition, high expression of miRNA-124 and decreased level of AMPAR are tightly associated with hippocampal demyelination and memory impairment in mice. And remyelination could restore the memory dysfunction and altered expression of miRNA-124 and AMPAR. Accordingly, it is possible that intervention of the potential miRNA-124/AMPA signaling may be a novel clue to improve memory performance in neurodegenerative diseases [98]. More importantly, miRNA-124 and miR-181a were engaged in cocaine-responsive plasticity through BDNF and downstream target, which played an important role in synaptic plasticity and addiction [99]. This data implies a promising possibility that a novel layer of miRNA-124 could be applied to drug rehabilitation in future.

## 8. Regulation of miRNA-125a and miRNA-125b in Synaptic Plasticity

Compared to other miRNAs at the synapse, miRNA-125a is abundant in the dendrites of hippocampal CA1 region and highly expressed in synaptoneurosomal fractions [66]. It has been showed that miRNA-125a is responsible for the translation of postsynaptic density (PSD-95), a core member of postsynaptic scaffold proteins with the function of managing synaptic strength and dendritic spine stabilization [100, 101]. Transfection of anti-miRNA-125a results in an upregulation of endogenous PSD-95 protein in the distal dendrites of hippocampal neurons, accompanied by significantly increased spine density and branching. And interfering with PSD-95 mRNA by specific siRNAs could reverse the altered spine morphology [101]. Further evidence reveals that the inhibition of miRNA-125a on PSD-95 is mediated by alternate G-quadruplex RNA conformations, which assist miRNA-125a to access into the binding site (G-rich region within 3′ UTR) of PSD-95 mRNA and form stable complex for interaction [22]. More intriguingly, the binding site of PSD-95 mRNA has been also shown to be directly recognized by FMRP, and phosphorylation of FMRP contributed to reinforcing the translational repression of PSD-95 induced by miRNA-125a [101, 102]. In addition, miRNA-125a is also involved in mGluR-mediated translation of local protein synthesis to modulate dendritic spine structure [101, 103].

The other homologous member of miRNA-125 family is miRNA-125b, which is also involved in synaptic plasticity [104–106]. It has been characterized that overexpression of miRNA-125b led to a significant increase in hyperphosphorylation of microtubule-associated protein tau and caused impairment of learning and memory. The activity was possibly mediated by the predicted targets of miRNA-125b including Bcl-2-like protein 2 (Bcl-W), dual specific phosphatase 6 (DUSP6), and protein phosphatase 1 catalytic subunit alpha isoform (PPP1CA) [105]. In addition, miRNA-125b induced downregulation of the essential synaptic glycoprotein synapsin-2 (SYN-2) is engaged in synaptic vesicles activity and synaptic transmitters trafficking in neuronal circuitry [104, 106].

## 9. Regulation of miRNA-188 in Synaptic Plasticity

Neuropilin-2 (Nrp-2), a receptor of semaphorin 3F, is capable of disturbing dendritic spine development and synaptic structure [107]. Lee et al. have reported that miRNA-188 served as an important modulator for synaptic plasticity by negatively targeting Nrp-2. In the study, induction of LTP led to decreased expression of Nrp-2 and increased level of synaptic activity-regulated miRNA-188, which could improve abnormal synaptic morphology and promote mEPSCs in hippocampus [108]. In addition, studies of neurodegenerative processes emphasized that miRNA-188-3p played a crucial role in the interaction of 2-arachidonoylglycerol (2-AG) metabolism and *β*-site amyloid precursor protein cleaving enzyme 1 (BACE1) expression in AD. On one hand, miRNA-188-3p expression was identified to be significantly reduced in APP transgenic mice, but it could be upregulated by 2-AG via peroxisome proliferator-activated receptor-*γ* (PPAR*γ*) and NF-*κ*B signaling pathway. On the other hand, high level of miRNA-188-3p in hippocampus gave rise to a set of biochemical and cognitive changes, including suppressions in BACE1 expression, A*β* synthesis and promotions in LTP, synaptic transmission, and spatial learning and memory. Moreover, validation of functional binding of miRNA-188-3p seed region in the 3′ UTR of BACE1 indicates that BACE1 acts as a downstream target of miRNA-188-3p governing synaptic plasticity and cognitive function [109].

## 10. Regulation of Other Specific miRNAs in Synaptic Plasticity

In addition to the above well-studied miRNAs, recent investigations show that other synapse-specific miRNAs are emerging to take a share in the process of plasticity modulation. miRNA-8 along with its multiple downstream target genes plays a critical role in neuromuscular synapse maturation at diverse stages in* Drosophila* [110, 111]. Synaptic cell adhesion molecules fasciclin III (FasIII) and neuroglian (Nrg) depend on miRNA-8 for accurate motor axon extension, suggesting that miRNA-8 has the capacity of regulating synaptic sites assembly at early stages of synaptic formation [110]. Besides, the regulation of miRNA-8 on enabled/vasodilator-stimulated phosphoprotein (Ena/VASP) family is essential for the presynaptic morphological modification required to match the substantial growth of postsynaptic targets at late stages of synaptic maturation [111].

Most recently, the study by Gao et al. reveals that miRNA-15a induced MeCP2 has a significant impact on the expression of BDNF, which subsequently plays a critical role in hippocampal dendritic branching and complexity [112]. The role of miRNA-19b in dendritic development and synaptic protein synthesis is correlated to the phosphoinositide-3-kinase/mammalian target of rapamycin (PI3K/mTOR) signaling that is triggered by phosphatase and tensin homolog (PTEN) [113]. Notably, miRNA-22 is suppressed by serotonin to mediate the regulation of cytoplasmic polyadenylating element binding (CPEB) genes in synaptic protein synthesis, which contributes to the maintenance of memory-related long-term synaptic plasticity [114]. Furthermore, miRNA-26a and miRNA-384-5p are consistently required for the maintenance of LTP and dendritic spine growth in CA1 region by targeting endogenous ribosomal S6 kinase 3 (RSK3) [115]. Inhibition of miRNA-26a can attenuate neurite outgrowth and neuronal morphogenesis via activation of PTEN [116]. In addition, Lippi et al. found that Arpc3 (subunit 3 in the actin-related protein 2/3 complex), which was targeted by miRNA-29a/b, modulated synapse structural plasticity via spine actin cytoskeleton remodeling [117].

Tap73, a transcription factor of p53 family, is shown to control the expression of miRNA-34a in hippocampal neurons, and this action is involved in the manipulation of synaptic proteins such as synaptotagmin-1 (Syt-1) and syntaxin-1A (Stx-1A) to regulate dendritic spine morphology and function [118, 119]. Meanwhile, miRNA-34a together with miRNA-193a and miRNA-326 synergistically drives the expression of activity-regulated cytoskeleton (Arc) associated protein in the processing of adaptive synaptic plasticity in response to external stimulation [120].

In addition, miRNA-128 regulates the dendritic arborization and intrinsic excitability of upper layer cortical neurons mediated by plant homeodomain finger 6 (PHF6) genes [121]. miRNA-137 manages dendritic spine patterning and plasticity-related protein synthesis in hippocampus via translational modulation of mind bomb 1 (Mib1) [122]. More importantly, miRNA-182 acts through cortactin and Rac1 to regulate synaptic protein synthesis in long-lasting plasticity which is the first characterization of miRNA-182 in lateral amygdala and provides a novel mechanism for modulating synaptic plasticity [123]. miRNA-191 and miRNA-135 contribute to the N-methyl-D-aspartate (NMDA) receptor-induced LTD, respectively, by targeting tropomodulin 2 (Tmod2) and complexin-1/2 [124].

## 11. Discussion 

The abundant evidence uncovers powerful regulatory role and mechanism of miRNA in various aspects of synaptic plasticity ranges from dendritic spine morphology and synaptic formation to plasticity-related protein synthesis (Table 1). The widespread miRNA-mediated regulation of synaptic plasticity is not only essential for neural development and physiological function but also involved in the pathogenesis and progression of multiple neurological disorders, such as AD, FXS, and epilepsy [27, 85, 89]. Furthermore, these findings lay favorable foundations for diagnosis and therapy of plasticity-related diseases. Actually, the identification of AD-related miRNAs in cerebrospinal fluid indicates that individual miRNAs may serve as promising biomarkers for AD diagnosis [86]. The regulation of miRNA-124 in cocaine-induced synaptic plasticity suggests that miRNA can be considered as a target for addiction therapy [99]. In addition, recent studies have showed that plasma miRNA-134 levels in mania patients treated with mood stabilizer lithium were significantly decreased, which was closely associated with synaptic pathology and clinical symptoms of manic [125]. These investigations expand our knowledge about miRNA contribution to the pathophysiology of neural plasticity and further improve the likelihood of applying miRNA-related strategies in clinical neurological diseases management. Currently, interfering with disease-related miRNAs and genes in miRNA cascade seems to be a promising miRNA-based therapeutic strategy. However, there is a great deal of problems that remain unsolved, one of which is lack of feasible targeted-delivery system to transport miRNA mimics or antagonists to specific miRNA in CNS* in vivo*. Recently, Hwang et al. reported that rabies virus glycoprotein-disulfide linked polyethylene imine could be used as a carrier to deliver neurogenic miRNA-124a across the blood brain barrier and make it accumulated in brain, but there was hardly any functional effect due to the degradation of miRNA in the processing of transportation [126]. As a result, improving the stability of transporter is another critical factor for excellent miRNA targeted-delivery system.

In conclusion, miRNA is a crucial regulator in synaptic plasticity. Combining high-throughput identification with functional assay of the interconnected miRNA network enables us to understand more information about the global effect of miRNAs from synaptic plasticity regulation to neurological diseases treatment. A deep insight into the mechanism through which the miRNA works for synaptic plasticity is significantly necessary before miRNA is applied to novel therapeutic intervention for neurological disorders. Thus, establishment of the entire framework of plasticity-related miRNAs will be a challenge in the near future.

## Figures and Tables

**Table 1:** Involvement of miRNA in synaptic plasticity.

miRNA	Target	Plasticity paradigm	References
miRNA-132		Regulated by CREB in activity-induced synaptic morphology, protein synthesis, and plasticity	[24, 25]
	p-CREB	Dendritic plasticity in TLE	[26]
	MFS	Dendrites morphology of CA3 region in spontaneous recurrent seizures	[27]
		Regulated by CREB in dendritic spine density and structure adaptation required for cognition capacity	[23, 28, 29]
	GR	Modulated by BDNF in synaptic formation and postsynaptic protein	[24, 30]
		Maturation of neuron and synapse in developing hippocampus	[31]
	p250GAP	RGC axonal sprouting and growth	[34]
	p250GAP	Activity-induced hippocampal dendrite spine morphogenesis and mEPSC and GluR1 positive spines	[35, 36]
	AChE	Stressful experiences induced neurite extension in cognitive and locomotion impairments	[37]
	Rac1	Synaptic actin-remodeling and spine morphological alteration in ionizing radiation	[38]
	p250GAP	Abnormal synaptogenesis and incorrect synaptic connectivity and function in PCB95 neurotoxicity	[40]
	p250GAP	Formation of stable dendritic spines and functional synapses in hippocampus	[39]
	MeCP2	Synaptic density and maturation in novel object recognition and memory retention	[42–44]
	MeCP2	Synaptic plasticity in RTT	[46, 48]
	MeCP2	Synaptic function in acute pain transduction and chronic pain formation	[47]
		Synaptic protein synthesis and dendritic spine structure related to FMRP	[49, 51]
	AMPAR	Neocortical *θ* burst-induced LTP and hippocampal synaptic transmission and plasticity	[56]
	IL-6, TSLP	Dendritic phenotype and synaptic formation in newborn neurons activity	[57, 58]
		Ocular dominance and visual cortex plasticity	[60–62]
		Modulated by REST/NRSF in synaptic plasticity and structural remodeling	[59]

miRNA-134		Regulated by SIRT1 in hippocampal LTP and long-term memory formation required for fear conditioning	[64]
		Regulated by DHX36 in plasticity-related genes and modification of dendritic spine size	[63]
	Pumilio2	Activity-induced hippocampal dendritic growth and excitatory synapse number	[65]
	Limk1	Dendritic spine size of neuron *in vitro* and dendritic arborization of cortical layer V pyramidal neurons* in vivo*	[66, 67]
	Limk1	Pyramidal neuron spine density in TLE	[74]
	DHHC9	Neuronal membrane trafficking and synaptic LTP and structure in specific interneurons by modification of H-Ras	[72]
	CREB	Synaptic plasticity in epileptic rat	[76]

miRNA-138	APT1	Dendritic spine size and synaptic transmission	[77]
		Episodic memory performance	[79]
	APT1	Synaptic protein synthesis in short-term recognition memory	[80]
	SIRT1	Axon growth in development and regeneration	[81]

miRNA-9	REST	Dendrites growth and synaptic transmission	[82]
	FOXG1	Affected by DLX5 in axon elongation and synaptic connectivity	[83]
	CAMKK2	Synaptotoxicity and dendritic spine loss in AD	[89]
	RhoG	Dendritic tree complexity and axonal branching	[93, 94]

miRNA-124	CREB	LTF in learning-related synaptic plasticity	[92]
	IQGAP1	Hippocampal LTP, long-term memory, and cognitive performance	[96, 97]
	AMPAR	Hippocampal demyelination and memory performance	[98]
	BDNF	Cocaine-induced plasticity and addiction	[99]

miRNA-125a	PSD-95	Synaptic strength and dendritic spine stabilization	[100, 101]
	GluR	Synaptic plasticity-related protein synthesis	[101, 103]

miRNA-125b	Tau	LTD and cognitive performance in AD	[105]
	SYN-2	Synaptic vesicles trafficking in neuronal circuitry	[104]

miRNA-188	Nrp-2	Dendritic spine development, synaptic structure, and mEPSC frequency	[108]
	BACE1	Regulated by 2-AG in hippocampal LTP, synaptic transmission, and cognitive function	[109]

miRNA-8	FasIII, Nrg	Assembly of synaptic sites at early stages of synaptic formation	[110]
	Ena/VASP	Presynaptic morphological modification at late stages of synaptic development	[111]

miRNA-15a	BDNF	Regulated by MeCP2 in hippocampal dendritic branching and complexity	[112]

miRNA-19b	PTEN	Synaptic development and synaptic protein synthesis	[113]

miRNA-22	CPEB	Memory-related long-term synaptic plasticity	[114]

miRNA-26a	PTEN	Synaptic plasticity and neuronal morphogenesis	[116]
	RSK3	Long-lasting synaptic and spine plasticity	[115]

miRNA-29a/b	Arpc3	Dendritic spine actin cytoskeleton remodeling and synaptic connectivity	[117]

miRNA-34a	Stx-1A, Syt-1	Regulated by Tap73 in dendritic spine morphology and synaptic function and plasticity	[118, 119]
	Arc	LTP, LTD, and adaptive synaptic plasticity	[120]

miRNA-128	PHF6	Dendritic arborization and intrinsic excitability of upper layer cortical neurons	[121]

miRNA-137	Mib1	Dendritic patterning and spine morphogenesis	[122]

miRNA-182	Rac1, cortactin	Long-lasting functional and structural plasticity	[123]

miRNA-191	Tmod2	Actin depolymerization in long-lasting dendritic spine remodeling	[124]

miRNA-135	Complexin-1/2	AMPAR exocytosis in prolonged spine structuring	[124]

Abbreviations in the table were illustrated in the text.
